# Targeting Metabolic Deregulation Landscapes in Breast Cancer Subtypes

**DOI:** 10.3389/fonc.2020.00097

**Published:** 2020-02-11

**Authors:** Erandi A. Serrano-Carbajal, Jesús Espinal-Enríquez, Enrique Hernández-Lemus

**Affiliations:** ^1^Computational Genomics Division, National Institute of Genomic Medicine, Mexico City, Mexico; ^2^Centro de Ciencias de la Complejidad, Universidad Nacional Autónoma de Mexico, Mexico City, Mexico

**Keywords:** cancer metabolism, pathway deregulation, breast cancer subtypes, therapeutic targets, steroid and fatty acid metabolism, purine metabolism

## Abstract

Metabolic deregulation is an emergent hallmark of cancer. Altered patterns of metabolic pathways result in exacerbated synthesis of macromolecules, increased proliferation, and resistance to treatment via alteration of drug processing. In addition, molecular heterogeneity creates a barrier to therapeutic options. In breast cancer, this broad variation in molecular metabolism constitutes, simultaneously, a source of prognostic and therapeutic challenges and a doorway to novel interventions. In this work, we investigated the metabolic deregulation landscapes in breast cancer molecular subtypes. Such landscapes are the regulatory signatures behind subtype-specific metabolic features. *n* = 735 breast cancer samples of the Luminal A, Luminal B, Her2+, and Basal subtypes, as well as *n* = 113 healthy breast tissue samples were analyzed. By means of a single-sample-based algorithm, deregulation for all metabolic pathways in every sample was determined. Deregulation levels match almost perfectly with the molecular classification, indicating that metabolic anomalies are closely associated with gene-expression signatures. Luminal B tumors are the most deregulated but are also the ones with higher within-subtype variance. We argued that this variation may underlie the fact that Luminal B tumors usually present the worst prognosis, a high rate of recurrence, and the lowest response to treatment in the long term. Finally, we designed a therapeutic scheme to regulate purine metabolism in breast cancer, independently of the molecular subtype. This scheme is founded on a computational tool that provides a set of FDA-approved drugs to target pathway-specific differentially expressed genes. By providing metabolic deregulation patterns at the single-sample level in breast cancer subtypes, we have been able to further characterize tumor behavior. This approach, together with targeted therapy, may open novel avenues for the design of personalized diagnostic, prognostic, and therapeutic strategies.

## 1. Introduction

Breast cancer is a complex, heterogeneous disease. Manifestations of this heterogeneity can be observed at the transcriptomic, molecular, or histological level (1). The origins of such manifestations can be traced back by looking at different levels of molecular control within the cells and tissues. The mechanisms behind gene expression, cell signaling, and metabolism are highly intertwined, and cross-regulation patterns appear (2, 3), which strongly determine the phenotypic variance observed in clinical practice (4–6). In fact, this broad variance in molecular metabolism in breast cancer constitutes, simultaneously, a source of prognostic and therapeutic challenges and a doorway to novel interventions (7–9).

In order to face the challenges posed by tumor heterogeneity, it is customary to classify or *subtype* tumors according to their feature similarity. One currently used classification method in breast cancer, which has been particularly useful for capturing biological functional features, is the so-called molecular subtyping (10). The default classification scheme in this regard is given by the PAM50 (10, 11) algorithm, which groups breast tumors into molecular classes or subtypes according to a gene-expression signature of 50 genes relevant to the patho-physiology of the tumor. These subtypes are *Luminal A, Luminal B, Her2+*, and *Basal*. Some authors include a fifth subtype, the so-called *Normal-like*, but its use is controversial, and its use has been in decline lately (12).

These subtypes have been able to capture relevant differences in the origin, prognosis, response to treatment, and relapse probability of breast tumors. In general, it is considered that luminal subtypes are less aggressive and have better prognosis and better response to treatment than non-luminal ones (11). However, under certain circumstances, Luminal B tumors may have a higher recurrence, less response to treatment, and worse long-term prognosis (13). This variation in response is not clear and is of the utmost importance for the understanding of the disease at the personalized level.

Genomic alterations (mutations, copy number variations, chromosomal aberrations) often derive into anomalous cell functioning, including deregulation of metabolism—an important emergent hallmark of cancer (14) via abnormal gene regulatory programs. Aberrant gene-expression patterns are currently studied using next-generation sequencing (NGS) techniques such as RNA-Seq.

The analysis of these gene deregulation signatures provides a comprehensive (genome-wide) approach to dig into the molecular basis of disease. In the case of tumor metabolism, one may argue that metabolomics and phospho-proteomics would be closer proxies to the actual underlying molecular mechanisms. However, despite important advances in experimental-omic techniques, comprehensive metabolomic mapping and fluxomics are still under-developed for the task of describing cellular metabolic processes comprehensively, although this should change in the upcoming years. Approaches to analyzing metabolic deregulation in cancer based on gene expression have been developed (15, 16). Those extensive studies used differentially expressed genes for more than 20 types of cancer to distinguish deregulated metabolic pathways. In both cases, specific pathways were identified as deregulated in particular types of cancer. However, those studies performed phenotype-specific analyses and did not focus on single-sample deregulation.

To overcome this issue, an appealing way to study deregulation of metabolism is by analyzing metabolism-related gene-expression signatures at a single-sample level. In this work, we used TCGA gene-expression data from 735 tumor samples (17, 18), classified according to their molecular signature, to investigate the pathway deregulation patterns for the four PAM50 molecular subtypes, to determine subtype-specific metabolic landscapes. We used a single-sample-based algorithm (19) to quantify metabolic anomalies. This algorithm provides a pathway deregulation score for each pathway at a sample level. For validation purposes, we used a 2,000-sample cohort (20) with the same pipeline. Analyzing metabolic deregulation patterns at the subtype and individual sample levels provides a means of characterizing tumor behavior with a view to designing personalized diagnostic, prognostic, and therapeutic strategies.

## 2. Materials and Methods

### 2.1. RNASeq Data Acquisition and Processing

Data were acquired from the Genome Data Commons Data Portal (https://bit.ly/2lJJrgi).

Briefly, 1,102 primary breast tumors and 113 normal solid tissues (normal solid tissue refers to healthy tumor-adjacent tissue taken from some of the tumors) samples were acquired and pre-processed to obtain *log*_2_ normalized gene-expression values (21). Data were pre-processed to eliminate intrinsic experimental biases (22).

#### 2.1.1. Integration

The following pipeline was already used and reported in Espinal-Enríquez et al. (21). Basically, an integrity check had to be carried out on raw expression files to ensure that all of them both had the same dimensions and provided TCGA identifiers before complementary annotation could be incorporated.

#### 2.1.2. Quality Control

The NOISeq R library was used for global quality control (23, 24). All samples reached saturation for the number of detected features at the corresponding sequencing depth. Global expression quantification for each experimental condition yielded a feature sensitivity >60% for 10 counts per million (CPM). **Bias** detection assessment showed the presence of gene length, %GC, and RNA patterns.

The EDASeq R library was used for batch-effect removal (25). Before normalization, genes with mean counts <10 were filtered, resulting in 17,215 genes, as suggested in Risso et al. (25). Different within/between **normalization** strategies were tested to remove bias.

Exploration of sample *log*_2_(normalized count) expression densities showed a consistent bi-modal pattern, corresponding to **noisy** lower-expressed genes and global sample behavior. Filtering out features with low counts (*CPM* < 10 cut-off) retained 15,281 genes, removing the undesired lower density peak. Finally, ARSyN R library was used for multidimensional noise reduction using default parameters (22).

#### 2.1.3. Subtyping

We classified the 1,112 breast cancer samples into the four molecular subtypes using the pbcmc R package (26), a variation of the PAM50 algorithm, which characterizes the assessment of the uncertainty in gene-expression-based classifiers (e.g., PAM50) based on permutation tests (12). Tumor samples with a non-reliable breast cancer subtype call were removed from the analysis. The number of removed samples was 377, giving a final number of 735 reliable samples.

### 2.2. Differential Expression Analysis and Pathway Discrimination

To determine overexpressed or underexpressed genes, we used the limma” R package (27), considering an absolute difference of Log_2_ FoldChange > 1 and a B-statistic > 5. The False Discovery Rate-adjusted *p*-value threshold was 10^−3^. Since the main goal of this work is to establish the extent of deregulation in the metabolism for each breast cancer sample/subtype, we kept 80 metabolic pathways present in the KEGG database (28) (the Pathifier algorithm needs a minimum number of molecules to be performed).

### 2.3. Pathway Deregulation Analysis

Metabolic pathway deregulation in each sample was quantified by using the Pathifier algorithm (19). This algorithm integrates the expression data of genes involved in a given metabolic pathway into a single deregulation value at the individual-sample level. the algorithm assigns a score between 0 and 1, called the Pathway Deregulation Score (PDS).” Values close to 0 correspond to samples whose expression levels are similar to controls (29). Samples with higher values present higher differences in expression levels compared to the control group. Pathifier quantifies the level of deregulation of a metabolic pathway in a single tumor sample by measuring the deviation of said sample from control behavior.

**Figure 1:**
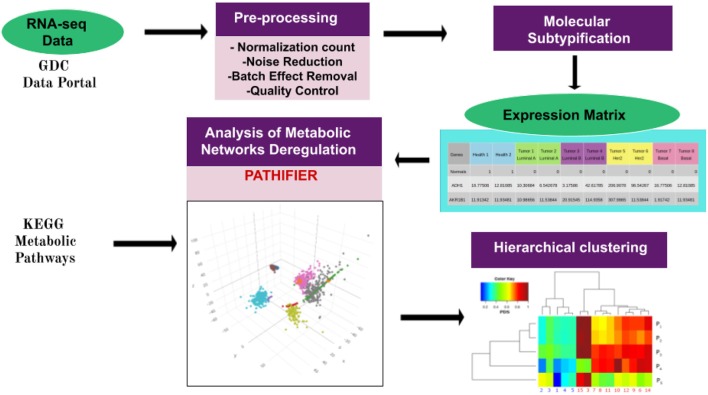
Pipeline of the work presented here. The workflow starts with data acquisition from the TCGA Genome Data Commons Data Portal. Pre-processing of gene-expression files was performed as in Espinal-Enríquez et al. (21). Breast cancer molecular classification was made by using the pcbcmc R package (12). Molecular subtype classification of normalized samples provides us with a gene-expression matrix, which is used to run the Pathifier algorithm (19). This algorithm assigns a Pathway Deregulation Score (PDS) to every metabolic pathway in each sample. The PDS is a score between 0 and 1. Here, 0 corresponds to the centroid of the control samples of a given pathway. The score increases according to the distance of the sample from this centroid along a principal curve, spanning the cloud of data points. The pathways used to perform Pathifier were obtained by filtering the metabolism-associated KEGG pathways. Finally, PDSs were grouped by using unsupervised hierarchical clustering. Hierarchical clustering modified from García-Campos et al. (30).

In some cases, a single sample with extreme gene-expression changes (majorly different from those of other samples) for genes in a given pathway may give rise to a really high (assigning PDS = 1 to that sample) score, making all other deregulated samples (with large but comparatively low gene-expression changes) close to zero, thus appearing to be minorly deregulated. In other words, several deregulated pathways/samples would be missing. In such cases, the outlier sample was removed from the analysis. Finally, an unsupervised clustering method was used to group samples with similar PDS. A graphical representation of the pipeline is presented here in Figure 1.

### 2.4. Identification of Potential Pharmacological Targets

Genes being commonly over/underexpressed in all breast cancer subtypes would suggest that there should be subtype-independent drugs. In order to assess this idea, we performed data mining on transcriptomic/drug data by using a previously developed (by our group) computational pipeline to find differentially expressed pharmacological targets of FDA-approved drugs (31) for those shared DEGs. This tool performs all possible combinations of differentially expressed targets and FDA-approved drugs in public pharmacological databases, as well as their two-drug interactions. So, for the more than 2611 drugs annotated in the DrugBank database and the 660 drugs annotated in PharmGKB, all subtype-specific differentially expressed genes were interrogated.

### 2.5. Validation

For validation purposes, we used 2,000 microarray samples from the METABRIC cohort (20), performed the same analysis with the already classified samples, obtained the single-sample PDS, and compared them with the TCGA cohort.

## 3. Results and Discussion

### 3.1. Subtype-Specific Deregulated Genes Are Associated With Characteristic Metabolic Pathways

**Figure 2:**
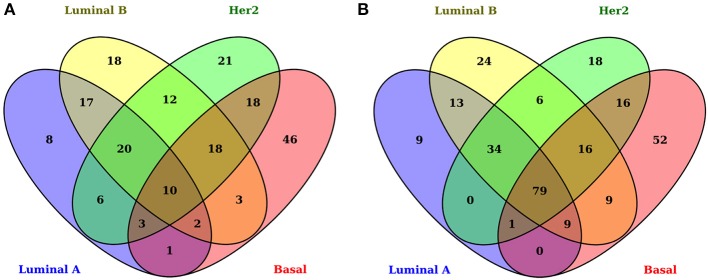
Differentially expressed genes associated with metabolism in breast cancer molecular subtypes. In these Venn diagrams, each ellipse corresponds to the DEGs appearing in each subtype. The left set **(A)** corresponds to overexpressed genes and the right set **(B)** to underexpressed genes. The number inside each subset represents the number of genes appearing in each subset. Notice that the center of both figures corresponds to the common DEGs for all subtypes. There are only 10 overexpressed shared genes, while, for the underexpressed subset, 79 genes appear.

As has been observed previously (1, 6), gene-expression signatures differ between all subtypes (Figure 2). The signatures presented here include only genes associated with metabolic pathways. Figure 2 shows the overexpressed and underexpressed metabolism-associated genes for each subtype in the form of a Venn diagram. It can be observed that all subtypes have a non-shared set of differentially expressed genes (DEGs) but also a small subset of shared deregulated genes.

By using |log_2_ *FoldChange*| > 1 and *B* − *statistic* > 5 as significance thresholds, the number of DEGs in all the tumors is 204 overexpressed and 287 underexpressed. The numbers of overexpressed and underexpressed genes for each subtype are very similar. Interestingly, the subset of shared overexpressed genes (*n* = 10) is substantially smaller than that of the underexpressed genes (*n* = 79). This difference between the number of shared underexpressed and overexpressed genes may be associated with the fact that some metabolic pathways are silenced or decreased in all subtypes; on the other hand, metabolic pathways with incremental activity are subtype-specific.

**Figure 3:**
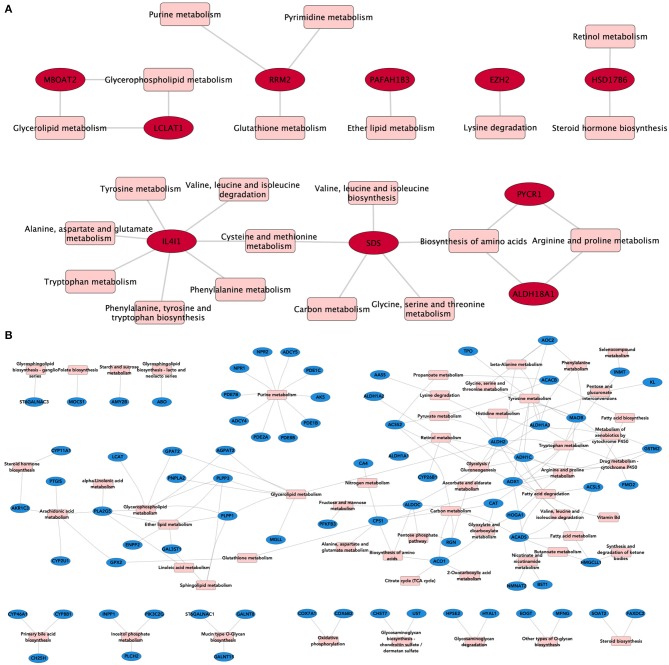
Metabolic processes associated with DEGs. In these bipartite network representations, overexpressed **(A)** and underexpressed **(B)** genes are depicted in red and blue, respectively. Metabolic processes are shown in pink squares. Links between both types of nodes appear if the gene is present in the corresponding pathway. For this network, we kept only those DEGs that appear in the four subtypes.

To evaluate whether shared overexpressed genes influence the regulation of metabolism, we associated them with the metabolic processes in which they participate. Figure 3 shows the relationships between the overexpressed genes (in red), and their associated metabolic processes (in pink) in the form of a bipartite network–a network composed by nodes of different nature, in this case, genes and pathways. Analogously, we constructed a network composed of the common underexpressed genes and their associated metabolic pathways.

As can be seen from the structure of the bipartite network, there are central molecules involved in several interrelated metabolic processes, giving rise to the so-called pathway-crosstalk events. This is a result of the utmost importance, since crosstalk phenomena have been associated with anomalous therapeutic responses and pharmacological resistance in breast cancer subtypes (32).

We can see, for instance, how the Interleukin 4-induced 1 gene (IL4I1) is the one with the most associated metabolic processes (*n* = 7), all related to amino acid biosynthesis (Figure 3A). This gene is often overexpressed in B-cell lymphomas (33) and has also been associated with cancer by promoting tumor growth and shaping the immune microenvironment in melanoma (34). Autoimmune suppression and the inhibition of *CD*8+ cells are also pro-tumor-associated mechanisms regulated by IL4I1 (35, 36). Such processes are ultimately linked to the metabolic activity of IL4I1 as a phenylalanine oxidase. Crosstalk events involving cross-regulation via IL4I1 and non-coding RNAs have also been reported to play a role in triple-negative breast cancer (37).

As can be observed from Figure 3B, common underexpressed genes participate collectively in specific metabolic processes, such as purine metabolism. This pathway provides the metabolites needed for survival and cell proliferation and DNA and RNA production (38). ATP and GTP are also products of this metabolic pathway.

Among the underexpressed genes, we may find ADCY genes (ADCY4 and ADCY5), which regulate the nucleotide proportion (39), AK5, which catalyzes degradation reactions of ATP (40), or PDE and NPR, which control the proportion of second messengers, strongly implicated in signal transduction (41).

The majority of these genes are involved in the formation/degradation of ATP. Since cell proliferation is a hallmark of cancer, we argue that underexpression of these genes may enable the tumors to avoid ATP/GTP degradation, thus providing energetic fuel to cell proliferation.

### 3.2. Metabolic Deregulation Patterns Are Characteristic of Each Breast Cancer Subtype

Once it has been shown that common deregulated genes induce regulation patterns in some metabolic processes, the remaining question is whether variations in the whole gene-expression signature correspond to changes in specific metabolic deregulation.

**Figure 4:**
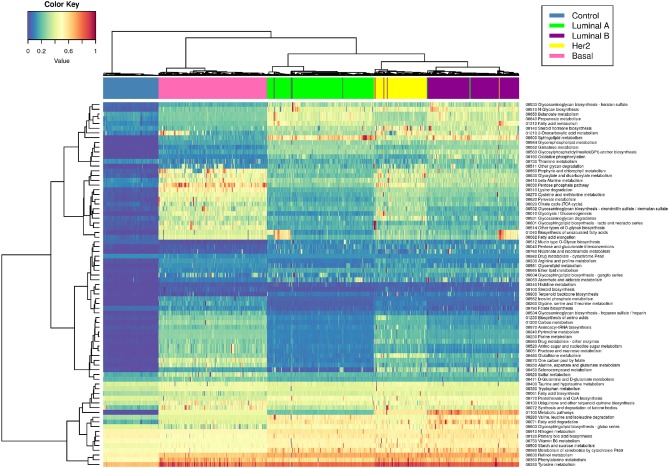
Metabolic deregulation in breast cancer subtypes. This heatmap shows the PDS for each sample (columns) in every metabolism-related pathway (rows). Blue color corresponds to lower PDS (close to 0), yellow color represents intermediate values, and red squares represent the samples with the highest scores. Dendrograms correspond to unsupervised hierarchical clustering for samples and pathways. The colored bar at the top of the heatmap represents the molecular subtype to which each sample belongs. Color code for molecular subtype is at the top right of the figure. Notice that the hierarchical clustering matches almost perfectly with the molecular subtypes (the color bars are practically separate from each other).

Figure 4 shows a heatmap of the PDS values (see methods) grouped by PDS similarity. Rows correspond to all pathways associated with metabolism, while columns correspond to samples. There are subsets of samples that present a similar metabolic deregulation among subgroups and differ from the other samples.

Interestingly, unsupervised hierarchical clustering of PDS coincides almost absolutely with the PAM50 classification. The colored bars in the upper part of the figure correspond to each subtype, and, as can be appreciated, each color of the bar is grouped together. This phenomenon reflects the high specificity of metabolic deregulation for each molecular subtype.

**Figure 5:**
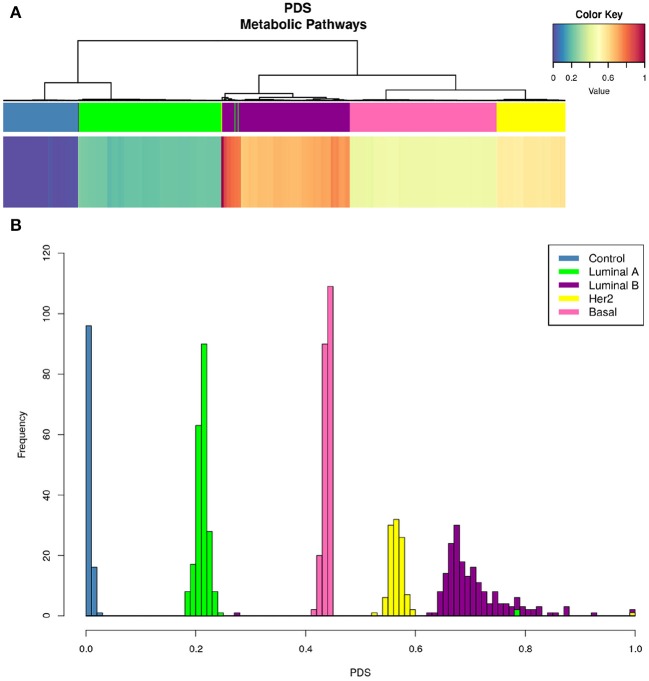
KEGG 00100 Metabolic pathways PDS in breast cancer subtypes. Upper figure shows the PDS for only one Kegg entry: 00100 Metabolic pathways. As in Figure 4, upper bar **(A)** shows that hierarchical clustering matches PAM50 subtypes even better than the whole set of pathways. From the color of the heatmap, it is possible to observe that deregulation per subtype follows this pattern: Luminal A, Basal, Her2+, and Luminal B. At the bottom **(B)**, we present the distributions indicating the frequency of PDS according to each subtype. Notice that the Luminal B histogram presents the largest variance, while the rest of phenotypes are, in general, confined to a narrow PDS range.

Figure 5 shows that only one KEGG pathway: 01100 Metabolic Pathways” contains the full set of 1,142 genes present in every metabolism-associated KEGG pathway. Hence, the PDS in this particular process summarizes (to a certain degree) information about the rest of the metabolic-related pathways. The PDS for each subtype again presents a subtype-specific behavior, but more widespread than in Figure 4.

The PDS values are different between all subtypes, but more importantly, it is clear to observe that Luminal B is the subtype with the highest PDS. This result was unexpected, since it is usually considered that the most aggressive and with worst prognosis is the Basal subtype (42). In this case, the order of deregulation is as follows: Luminal A, Basal, HER2+, and, finally, Luminal B (Figure 5). From the PDS distributions, it can be noticed that the Luminal B subtype has the highest values but also the largest variance between samples. The rest of the subtypes are highly concentrated in a narrow range of PDS.

Previous reports have also analyzed the relationship between transcriptional deregulation and metabolic changes in cancer (15, 16). From these studies, some commonalities and differences arise. The work of Rosario uses differentially expressed genes for several phenotypes, breast cancer subtypes included. There, a score is based on LogFoldChange and adjusted *p*-values, measures that have not been derived with pathway-level assessment in mind, in contrast with the PDS, which is a specific pathway-level measure.

**Figure 6:**
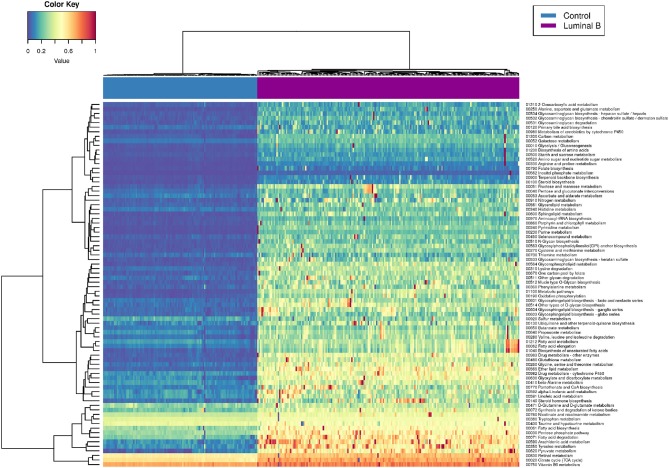
Luminal B metabolism PDS. This heatmap shows the deregulation of KEGG metabolism-related pathways in Luminal B tumors. Some samples are highly deregulated in a small subset of pathways.

Regarding commonalities, metabolic pathways are found to be differentially regulated in all subtypes in both manuscripts, in spite of the different approaches to pathway scoring. Purine and retinol metabolism are also found to be highly deregulated in both studies, particularly in the Luminal B and Basal subtypes. Interestingly enough, the Luminal B and Basal subtypes are the most deregulated phenotypes in both studies. This is reflected in Figure 6d from Rosario's paper and in Figure 4 in our manuscript.

Another point in common between both studies is the coincidence of the Citric acid cycle as a unique pathway observed in the Basal subtype, with the TCA cycle found in our Basal samples (Figure 4). Interestingly, the categories reported in Figures 6d–f of Rosario's paper correspond to those of the Reactome database and not the ones described in the KEGG database. This is relevant since the categories are similar but not identical. This may be an additional source of some apparent discrepancies between Rosario's results and ours.

Regarding differences, Rosario et al. found different pathway scores for the Basal and Luminal A subtypes. However, as can be seen from Figure 6C of Rosario's paper, the low specificity of the average gene-expression Z-scores results in a non-conclusive depiction, as it is hard to distinguish signal from background noise. This is also reflected in the density plot of the Figure 6C heatmap. Additionally, the hierarchical clustering on top of the heatmap reflects a large degree of heterogeneity, resulting from the broad variance of the average gene-expression profiles. However, a clear phenotypic fingerprint of basal tumors is actually captured in terms of average gene-expression profiles, likely due to a reduced heterogeneity in these tumors.

### 3.3. Luminal B Tumors Present Higher Pathway Deregulation Scores

Figure 6 represents the PDSs for the Luminal B subtype only. It can be observed that several metabolic processes are highly deregulated (reddish rows), such as is the case of pyruvate metabolism, tyrosine metabolism, fatty acid degradation, and the pentose phosphate pathway.

In some cases, only a small subgroup of samples presents high PDSs (scattered red pixels), which in turn reflects the intrinsic heterogeneity of samples, even if they belong to the same subtype. In the following, we will make some remarks regarding the most deregulated metabolic pathways observed in Luminal B tumors.

Pyruvate-related metabolic reprogramming has been associated with metastatic potential and treatment resistance in cancer (43). Pyruvate is a central metabolite for glucose, lactate, lipids, and amino acids. In breast cancer, liver-metastatic breast cancer cells exhibit a unique metabolic program compared to bone- or lung-metastatic cells, converting glucose-derived pyruvate into lactate, with a concomitant reduction in glutamine. This metabolic reprogramming results in a higher metastatic potential (44). Deregulation of fatty acid metabolism is crucial for malignant transformation in breast cancer. Proteins involved in the synthesis and oxidation of fatty acids play a pivotal role in the proliferation, migration, and invasion of breast cancer cells. Additionally, it has been shown that molecular subtypes display specific fatty acid metabolism (45). Deregulation of fatty acid metabolism has been associated with non-luminal tumors. Luminal subtypes rely on a balance between de novo fatty acid synthesis and oxidation as sources for biomass and energy. On the other hand, triple-negative basal breast cancer often uses exogenous fatty acids. In terms of targeted, personalized therapy, it is desirable to take such differences into account. In the case of the pentose phosphate pathway (PPP), it has been shown that PPP-associated proteins, such as 6PGL, 6PGDH, or NRF2, are not differentially expressed among breast cancer subtypes but are overexpressed relative to control samples (46). Glucose 6-phosphate dehydrogenase G6PD has been closely associated with prognosis in Basal tumors (47). It has been demonstrated that G6PD silencing increases the glycolytic flux, reduces lipid synthesis, and increases glutamine uptake in breast cancer cells. This effect has also been strongly related to poor prognosis (48). Her2-positive Luminal B tumors present overexpression of G6PDH (49). However, even if the presence of PPP-related proteins in Luminal B breast cancer has been established, a global analysis of this pathway is still lacking.

As we have said, the Luminal B subtype is the one with the highest metabolic deregulation. It is known that, in the long-term, the Luminal B subtype presents higher drug resistance, metastasis, and relapses (50, 51). This could be, in part, due to the individual heterogeneity at the gene-expression level. The metabolic deregulation in this subtype could also underlie drug resistance.

To our knowledge, a profound study regarding metabolism in the Luminal B subtype is still necessary. However, we suggest that the long-term malignancy and poor prognosis of the Luminal B subtype are due, in part, to global metabolic deregulation more than to any single-molecule alteration. Further analyses in this regard are required to assess the metabolic deregulation patterns observed here with higher accuracy.

### 3.4. Purine Metabolism as a Potential Target in All Breast Cancer Subtypes

For the more than 2,611 drugs annotated in the DrugBank database and the 660 drugs annotated in PharmGKB, all subtype-specific differentially expressed genes were matched. The top 20 identified potential pharmacological targets obtained by the pipeline performed in Mejía-Pedroza et al. (31) are reported in Table 1. It contains those drugs that inhibit overexpressed genes. Table 2 lists those drugs that activate underexpressed ones.

**Table 1:** Overexpressed genes with FDA-approved inhibitors to regulate purine metabolism.

**Search term**	**Drug**	**Interaction type**
RRM2	FLUDARABINE PHOSPHATE	Inhibitor
RRM2	GALLIUM NITRATE	Inhibitor
RRM2	CLADRIBINE	Inhibitor
RRM2	CLOFARABINE	Inhibitor
RRM2	FLUDARABINE	Inhibitor
RRM2	GEMCITABINE	Inhibitor
RRM2	HYDROXYUREA	Inhibitor
RRM2	MOTEXAFIN GADOLINIUM	Inhibitor
RRM2	TEZACITABINE	Inhibitor
RRM2	GEMCITABINE HYDROCHLORIDE	Inhibitor
EZH2	CHEMBL3287735	Inhibitor

**Table 2:** Underexpressed genes with FDA-approved activators to regulate purine metabolism.

**Search term**	**Drug**	**Interaction type**
ACACB	METFORMIN	Activator
NPR1	ATACIGUAT	Activator
PDE1C	BEPRIDIL	Activator
PDE2A	CHEMBL395336	Activator

As can be observed in Table 1, RRM2, which participates in purine, pyrimidine, and glutathione metabolism, is the most targeted gene. EZH2, involved in lysine degradation, is another target that may be inhibited.

It is worth noticing that this computational tool provides all FDA-approved drugs that target a list of molecules, together with the effect that is produced in the target. Supplementary Tables 1, 2 contain comprehensive lists of drugs and their targets for commonly overexpressed and underexpressed breast cancer genes.

In the case of underexpressed genes, three of the four targets of activator drugs participate in purine metabolism: NPR1, PDE1C, and PDE2A. This result appears to be relevant in terms of the potential therapeutic options that breast cancer patients may have. There is a common deregulated metabolic pathway (purine metabolism) that can be targeted by specific drugs that have activator and inhibitory actions over underexpressed/overexpressed genes, respectively.

### 3.5. Deregulation of Metabolism Is Consistent in a Different Cohort

We performed a comparison with data from METABRIC (20), another large breast cancer cohort study. Our validation analysis shows a separation between groups as in the discovery group. A heatmap of the validation cohort is presented in Supplementary Figure 1, and the distribution of PDS in the METABRIC dataset is presented in Supplementary Figure 2. Some of our findings replicate those of METABRIC, although there were also differences, some of which may be attributable to METABRIC being a microarray-based experimental approach, whereas TCGA included data from RNA-sequencing experiments.

## 4. Conclusions

Heterogeneity is a crucial factor that impedes the understanding, diagnosis, and treatment of breast cancer tumors. Manifestations of this heterogeneity can be observed at the genomic, histological, or clinical level. In this work, we have provided another instance of this heterogeneity: metabolic deregulation.

Each breast cancer subtype has its own pattern of deregulation in metabolism, with Luminal B having the highest deregulation scores. This subtype presents alterations to metabolic processes such as pyruvate metabolism, tyrosine metabolism, fatty acid degradation, and the pentose phosphate pathway.

To our knowledge, this is the first time that a single-sample-based pathway analysis in breast cancer subtypes has been performed to identify differences in metabolic regulation. At the same time, this work has allowed us to design a common therapeutic FDA-approved scheme to regulate purine metabolism, independently of the subtype. With this kind of approach, it is possible to determine global deregulation patterns while, at the same time, finding individual signatures that may represent a further step toward personalized medicine.

## Data Availability Statement

The datasets generated for this study can be found in the (Genomic Data Commons Data Portal).

## Author Contributions

EH-L and JE-E contributed to the conception and design of the study. ES-C collected, organized the database, preprocessed the data, performed the computational analysis, and performed results visualization. ES-C, JE-E, and EH-L discussed and contextualized the results. JE-E and EH-L wrote the first draft of the manuscript. All authors contributed to manuscript revision, read, and approved the submitted version.

### Conflict of Interest

The authors declare that the research was conducted in the absence of any commercial or financial relationships that could be construed as a potential conflict of interest.

## Supplementary Material

Supplementary Figure 1 PDS heatmap of validation cohort. This heatmap showing the deregulation of KEGG metabolism-related pathways in the METABRIC cohort. Color code is the same as in the figures in the main manuscript.

Supplementary Figure 2 Distribution of PDS in the validation cohort. Distributions indicating the frequency of PDS according to each subtype in the METABRIC cohort.

Supplementary Table 1 Comprehensive lists of drugs and their targets for commonly overexpressed breast cancer genes.

Supplementary Table 2 Comprehensive lists of drugs and their targets for commonly underexpressed breast cancer genes.
