# Supplementary figures and images for: Targeting Metabolic Deregulation Landscapes in Breast Cancer Subtypes
Source: Front Oncol. 2020 Feb 11;10:97. doi: 10.3389/fonc.2020.00097 (PMC7026677; doi:10.3389/fonc.2020.00097)

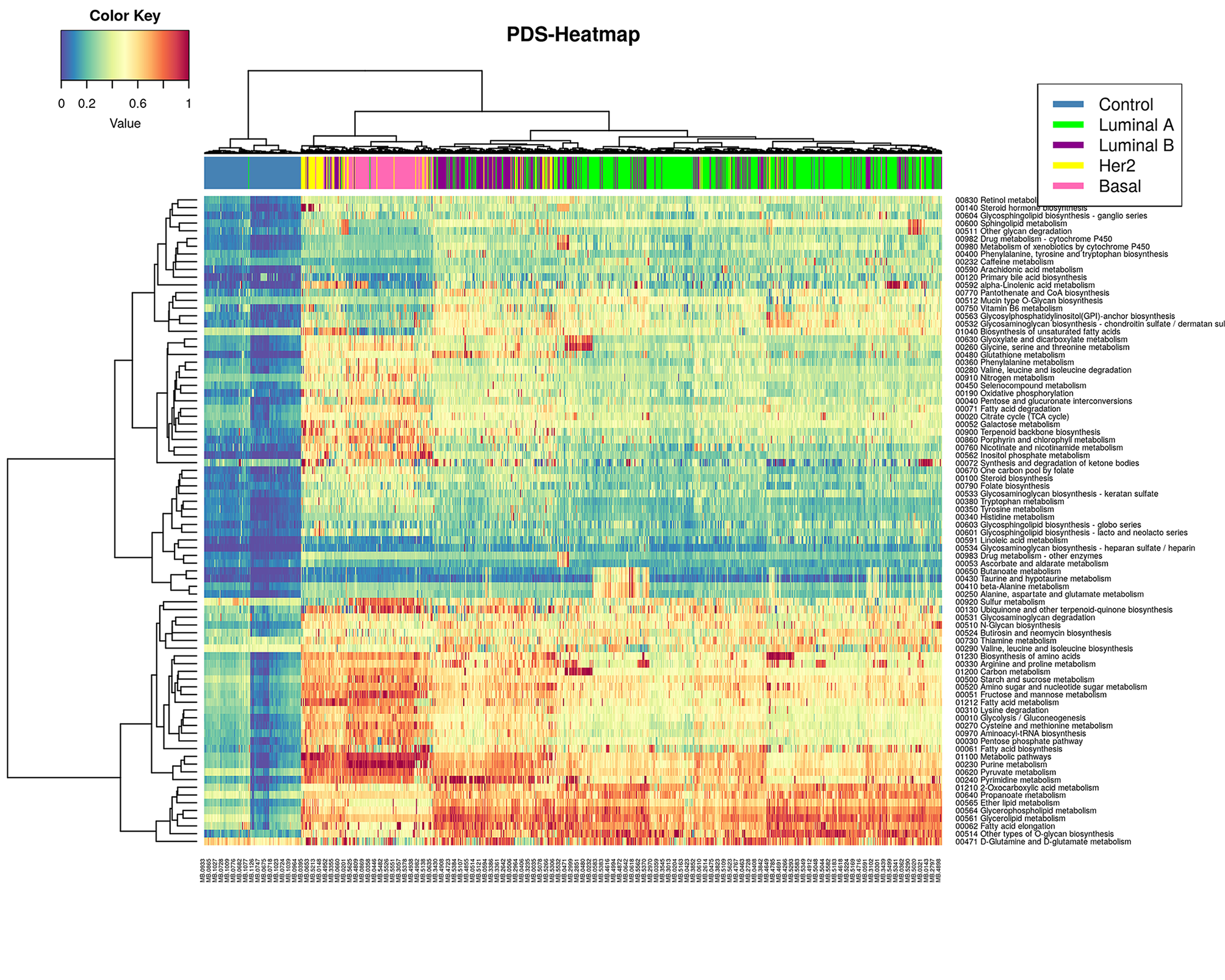

Supplement: Supplementary Figure 1 — PDS heatmap of validation cohort. This heatmap showing the deregulation of KEGG metabolism-related pathways in the METABRIC cohort. Color code is the same as in the figures in the main manuscript. [file Image_1.TIFF]

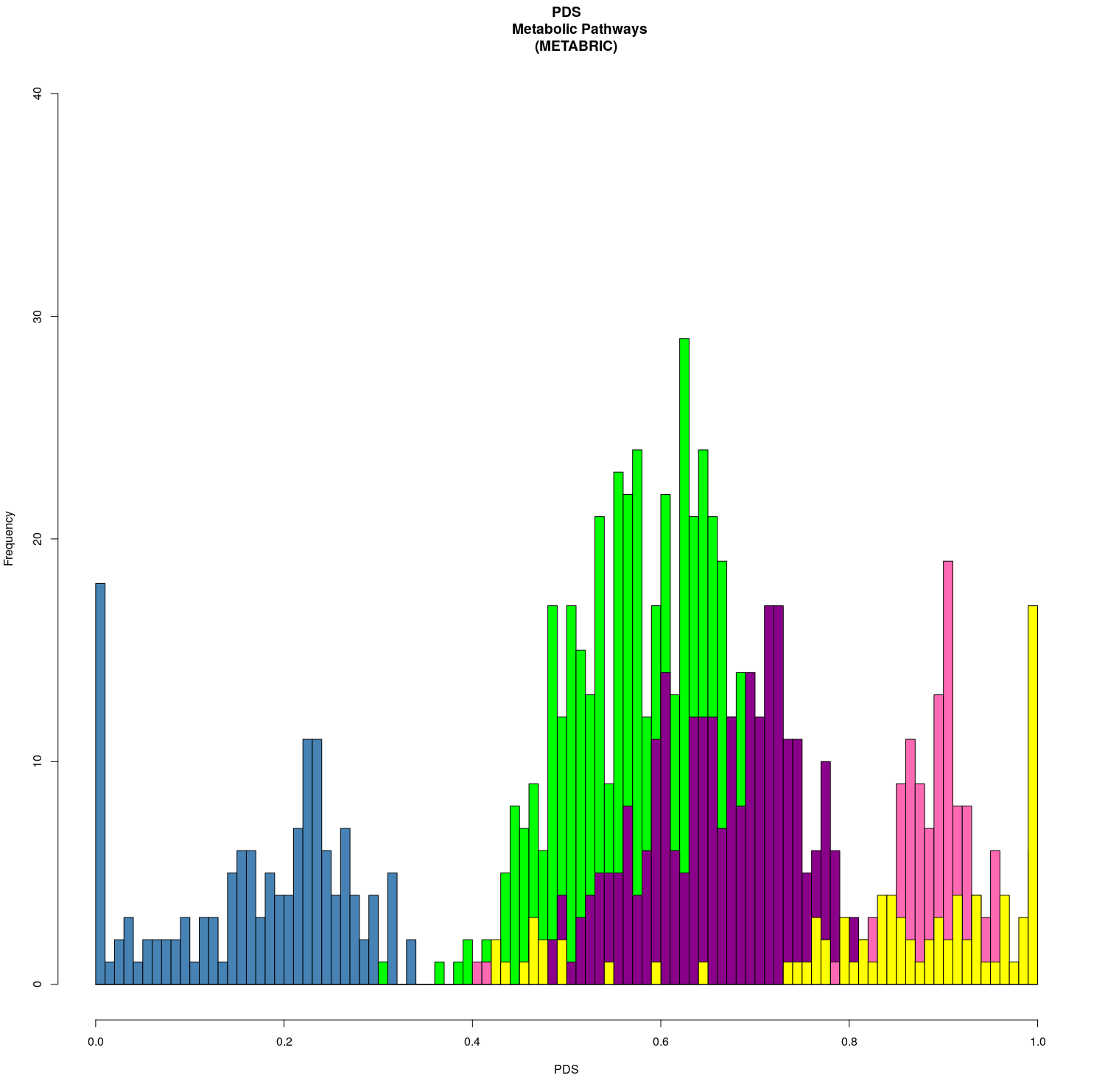

Supplement: Supplementary Figure 2 — Distribution of PDS in the validation cohort. Distributions indicating the frequency of PDS according to each subtype in the METABRIC cohort. [file Image_2.TIFF]
